# Calcifying fibrous tumor

**DOI:** 10.4322/acr.2021.400

**Published:** 2022-09-20

**Authors:** Hanan Elsarraj, Ameer Hamza

**Affiliations:** 1 The University of Kansas Medical Center, Department of Pathology and Laboratory Medicine, Kansas City, Kansas, USA

**Keywords:** Gastrointestinal Stromal Tumors, Intestinal Polyps, Neoplasms, Fibrous Tissue

## Abstract

Calcifying fibrous tumor is a rare benign mesenchymal neoplasm. The etiology and pathogenesis of this tumor are uncertain. It has wide anatomical distribution. The tumor is most commonly found in the soft tissues of the extremities in younger individuals. However, in middle-aged patients, it tends to affect the visceral locations more commonly. In visceral location, it can mimic aggressive lesions clinically. The purpose of this report is to describe a case of calcifying fibrous tumor in a 71-year-old female with a history of breast carcinoma who was found to have an incidental small bowel mass on her follow-up. Clinically and radiologically, the mass was suspicious for either metastatic disease or gastrointestinal stromal tumor.

The patient underwent open small bowel resection, and a 6.5 cm segment of the small bowel was sent to pathology. Grossly, a 2.0 cm tan-pink smooth round submucosal polyploid mass protruding into the lumen, mimicking a gastrointestinal stromal tumor, was identified. The tumor was hard and serially sectioned to reveal a white, calcified cut surface. Microscopically, the tumor appeared hypocellular and composed of scant spindle cells embedded in a dense, hyalinized and calcified collagenous stroma. Immunohistochemical stains for pan-cytokeratin, DOG1, desmin, S100, CD34, and MUC4 were negative, and a diagnosis of the calcifying fibrous tumor was rendered.

This case provides a rare gross specimen image of calcifying fibrous tumor and highlights the importance of knowledge of rare entities in providing an accurate diagnosis for entities that can mimic other lesions.

## INTRODUCTION

Calcifying fibrous tumor (CFT) is a rare benign mesenchymal neoplasm. The etiology and pathogenesis of this tumor are uncertain. It was first described in 1988 by Rosenthal and Abdul-Karim^1^ and was termed childhood fibrous tumor with psammoma bodies. The World Health Organization (WHO) established the name “Calcifying Fibrous Tumors” for this lesion in 2002.^2^ CFT affects patients in a wide age range and has a wide anatomic distribution. In a review of 157 cases, a slight female predominance (56%) was noticed, with a mean age of 33.5 years (range 5 weeks to 84 years).^3^ Anatomical distribution includes soft tissues, neck, gastrointestinal tract, mesentery, peritoneum, mediastinum, and adrenal gland. Within the gastrointestinal tract, the tumor is frequently submucosal, and the most common sites are the stomach and small intestine.^4^ Herein we describe a case of calcifying fibrous tumor of the small bowel.

## CASE REPORT

**Figure 1:**
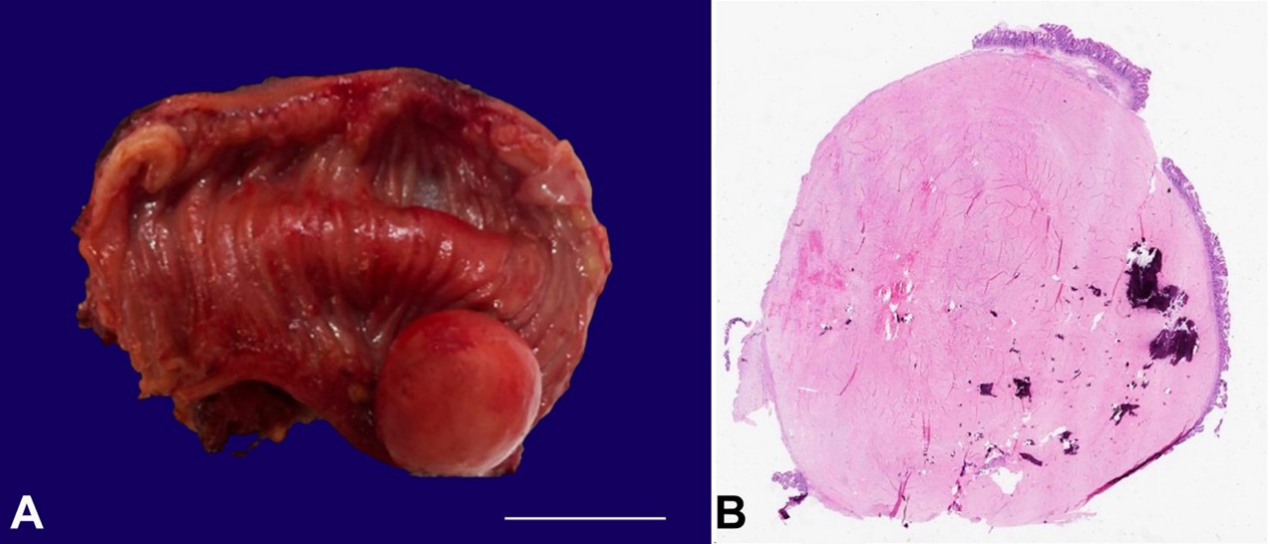
**A** – Gross appearance of the tumor in the segment of the small bowel (scale bar= 2 cm); **B** – Photomicrograph demonstrating whole-mount view of the tumor (H&E 5x).

**Figure 2:**
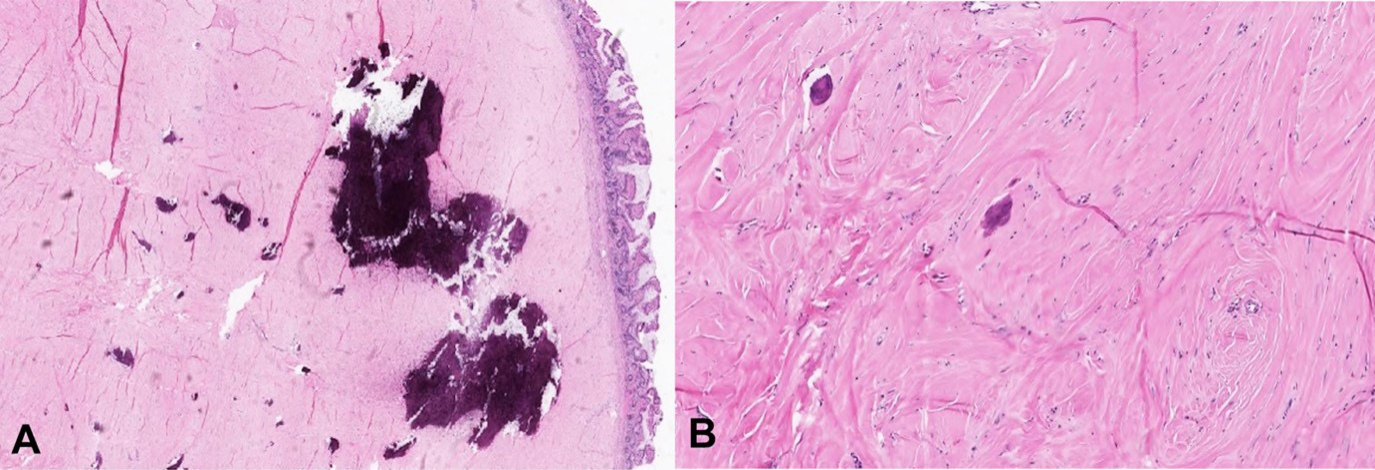
**A** – Medium power photomicrograph showing the tumor in relation to overlying normal mucosa (A- H&E 10x); High power photomicrograph showing scant spindle cells embedded in a dense, hyalinized and calcified, collagenous stroma (B- H&E 20x).

A 71-year-old female was diagnosed with invasive ductal carcinoma of the left breast two years ago. The tumor was histologic grade2, ER+ / PR+ / HER2-. The patient underwent a mastectomy followed by adjuvant endocrine therapy. She was found to have an incidental small bowel mass on a CT scan during her follow-up. The mass was located 50-60 cm from the ileocecal valve. Clinically and radiologically, the mass was suspicious for either metastatic disease or gastrointestinal stromal tumor. The patient underwent open small bowel resection, and a 6.5 cm segment of the small bowel was sent to pathology. Grossly, a 2.0 cm tan-pink smooth round submucosal polyploid mass protruding into the lumen was identified (Figure 1A). The tumor was hard and serially sectioned to reveal a white, calcified cut surface. Microscopically, the tumor appeared hypocellular and composed of scant spindle cells embedded in a dense, hyalinized and calcified, collagenous stroma (Figure 1B, and Figure 2). No cytologic atypia or mitotic activity were identified. There was no inflammatory infiltrate, except for occasional scattered lymphocytes. Immunohistochemical stains were performed to rule out differential diagnoses, and the spindle cells were found to be negative for pan-cytokeratin, DOG1, desmin, S100, CD34, and MUC4. A final diagnosis of calcifying fibrous tumor was rendered.

## DISCUSSION

In the gastrointestinal tract, CFTs are usually discovered incidentally; however, they can also manifest as ulcers if involving the stomach or with bowel obstruction when present in the small intestine. Abdominal or epigastric pain is another common presenting complaint. Grossly, gastrointestinal CFTs are submucosal and range in size from 0.5 to 11.0 cm, with an average size of 2.6 cm.^4^ They are often well-demarcated, unencapsulated, spherical to lobulated masses with a firm to hard, tan-white to gray cut surfaces.

Microscopically, CFT comprises a paucicellular fibroblastic proliferation with bland spindle cells embedded in dense collagenous tissue, variable degree of lymphocytes, and scattered dystrophic or psammomatous calcification. The differential diagnosis includes inflammatory myofibroblast tumor (IMT), leiomyoma, IgG4-related disease, fibromatosis, desmoplastic fibroblastoma, fibroma of the tendon sheath, low-grade fibromyxoid sarcoma, and gastrointestinal stromal tumor (GIST).^5^

CFTs can be histologically very similar to IMT (specifically sclerosing type) as both entities are paucicellular with dense fibrous stroma and can have lymphocytic infiltrate. The two entities can be differentiated by the presence of ALK gene rearrangements or ALK-1 immunohistochemical stain positivity in IMT; however, it is noteworthy that only ~50% of IMTs are positive for ALK. Additionally, IMTs are frequently SMA positive and show variable expression of desmin.

The cytological features of leiomyoma are sufficient to differentiate it from CFT; however, leiomyomas can also be paucicellular and hyalinized. In these cases, desmin, SMA, and caldesmon can help the distinction.

CFTs may contain IgG4 plasma cells, but it can be distinguished from IgG4-related disease, which depicts dense storiform fibrosis, obliterative phlebitis, and a much higher number of IgG4 positive plasma cells.

Grossly, fibromatosis is poorly circumscribed and microscopically has sweeping fascicles of elongated spindle cells. Additionally, it lacks prominent inflammation and calcifications and frequently shows nuclear positivity with beta-catenin stain. Both desmoplastic fibroblastoma and fibroma of the tendon sheath lack the distinctive inflammation and calcification that are found in CFTs.

Low-grade fibromyxoid sarcoma usually occurs in the proximal extremities and trunk. It classically shows hypocellular fibrocollagenous and cellular myxoid zones and diffuse, strong MUC4 positivity.

GISTs are generally more cellular and lack inflammation, hyalinized collagen, and calcifications. They are frequently positive for CD117 and DOG1, which are negative in CFT.

Mehrad et al.^6^ performed whole-exome sequencing on three specimens of pleural calcifying fibrous tumors and found mutations in ZN717, FRG1, and CDC27 genes. They also identified copy number losses on 8 chromosomes and a large loss common to all cases on chromosome 6.

Management of CFTs depends on the location and treatment of the symptoms. They are considered benign and cured by local resection of the tumor.^4^ Recurrence is known to occur. In a study by Nascimento et al.^7^ local recurrence rate was 20%. Malignant transformation has not been reported in the literature, and the overall prognosis is excellent regardless of the anatomical location.^5^
